# Cross-resistance studies on 1,6-dibromo-dideoxy-D-mannitol(DBM)-resistant Yoshida S.C. sarcoma.

**DOI:** 10.1038/bjc.1967.40

**Published:** 1967-06

**Authors:** E. Csányi, M. Halász


### CROSS-RESISTANCE STUDIES ON 1,6-DIBROMO-DIDEOXY-D-MANNITOL(DBM)-RESISTANT YOSHIDA S.C. SARCOMA

E. CSÁNYI AND MARIA HALÁSZ

*From the Research Institute for Pharmaceutical Chemistry, Budapest, Hungary*

Received for publication November 29, 1966

THE extensive and detailed studies made on various resistant experimental tumours offer considerable help in the recognition of the mechanism of anti-cancer activity.

Similarly to the antimetabolite-resistant tumours a number of strains have been developed showing varying degrees of resistance to the biological alkylating agents. Hirono (1954) made one of the first investigations of the resistance with N-mustard-N-oxide. Since then many resistant sublines have been developed and reported in the last years.

Ujhazy and Winkler (1965) have prepared and studied a Yoshida sarcoma line resistant to nitrogen mustard and Gáti and Kellner strains which are resistant to Degranol (Gáti, 1965). Myura (1961) and Moriwaki (1963) have described mustard-resistant ascites hepatoma strains and have demonstrated the acquired resistance of these sub-strains to alkylating cytostatics. Most frequently the resistant strains are developed by adaptation. Animals are treated with non-curative doses of a cytotoxic agent throughout a large number of passages of the originally sensitive tumour, when an increasing degree of resistance of the tumour to the chemotherapeutic agent develops. This seems to be a fairly reliable method and resistance developed in this way is usually hereditary.

Sakurai (1963) suggested an *in vitro* method for the study of resistance. According to his data, neoplastic cells incubated with N-mustard displayed resistance of several hundredfold towards this agent after only one or two periods of incubation with the drug.

The degree of resistance is usually determined by *in vivo* experiments, though Moriwaki (1963) suggested the use of an *in vitro* method.

The anti-neoplastic and haematologic effects of 1,6-dibromo-1,6-dideoxy-D-mannitol (DBM), Myelobromol were first described in 1961 (Csanyi *et al.* 1961, 1964, 1965). Although the chemical structure of this compound differs from that of other known biological alkylating agents, earlier experiments have not shown that it has a different mechanism of action (Institoris *et al.*, 1964*b*, Horvath *et al.*, 1966). The compound has been applied clinically under the name of Myelobromol, mainly for the treatment of chronic myeloid leukaemia (Sellei, 1963, Sellei and Eckhardt, 1963; Eckhardt *et al.*, 1963, 1964). Good results have been obtained even in cases resistant to other cytostatic agents (Myeleran, Mannitol-Myleran, X-ray therapy etc.).

To gain a more thorough insight into the mechanism of action of DBM, a DBM-resistant Yoshida-sarcoma line has been developed and a number of cross-resistance tests have been carried out to demonstrate the existence of, or lack of, cross-resistance to biological alkylating agents of various types to antibiotics, to antimetabolites and to mitotic poisons.

The new tumour line developed from Yoshida-sarcoma has been named D-Yoshida sarcoma.

### METHODS

The first experiment has been done in order to produce DBM-resistant Yoshida sarcoma strains by Sakurai's *in vitro* method (1963). The recommended concentration of $5 \times 10^{-4}$ M/ml. was, however, too high in the case of DBM, as this dose resulted in the complete inhibition of " takes ". Repeated use of single lower doses, on the other hand, produced no resistance whatsoever. Contrary to Sakurai's experiences with nitrogen mustard, where resistance developed after only 1 or 2 incubation periods, a certain degree of resistance was observed only after 15 incubation passages.

The preparation of a more highly resistant tumour strain was as follows:

From the fifth day after subcutaneous implantation of the Yoshida sarcoma the rats were treated on three successive days with 30 mg. per kg. of DBM i.p. The tumours were transplanted one week after the end of the treatment. In the following periods up to the eighth generation the beginning of the DBM treatment was gradually brought closer to the time of implantation, also, the dose was raised gradually to 70 mg. per kg. Development of resistance was first demonstrated clearly with the eighth treated generation of the tumour. From the eighth to the fifteenth generation the rats were treated with $6 \times 70$ mg. per kg. of DBM i.p. and the tumours were transplanted every eighth day. The degree of resistance was determined from the 20th and 40th generation; cross-resistance tests were carried out from the 20th to 25th generation.

The animals used in the experiments were female CB Wistar rats with a body weight of 150–200 g. For tumour implantation the usual subcutaneous trocar technique was used. The water soluble agents were dissolved in physiological saline, the insoluble compounds suspended in Tween-80 and all were administered intraperitoneally. The experiments were evaluated by using the weight of the tumours on the eighth or ninth day.

### RESULTS

### *Presence and degree of resistance*

From the eighth treated generation the difference between the inhibitory effect of 100 mg./kg. DBM obtained on sensitive and resistant tumours growing in the same animals was already significant. The degree of resistance was first determined after the 20th generation. The results are summarized in Table I.

### *The effect of DBM on sensitive and resistant 20th generation Yoshida s.c. sarcoma*

According to the data in the Table I showing the high degree of acquired resistance, a significant inhibition was obtained only with subtoxic doses of DBM on D-Yoshida sarcoma. For the cross-resistance tests the doses of the agents were chosen in such a way as to produce the maximum possible effect on sensitive Yoshida sarcoma.

The agents used for the test belong to the following classes:

1. Biological alkylating agents:
   (a) nitrogen mustards
   (b) ethyleneimines
   (c) methanesulphonates
   (d) halogenated sugar alcohols

2. Antimetabolites (a) folic acid antagonists
   (b) purine, pyrimidine antagonists
3. Mitotic poisons
4. Antibiotics.

TABLE I.

| Dose mg/kg 5 × i.p. | Yoshida s.c. sarcoma Weight of tumour mg. | Inhibition % | D-Yoshida s.c. sarcoma Weight of tumour mg. | Inhibition % | Change in body-weight % |
|---|---|---|---|---|---|
| Control | 2110 | — | | | + |
| 2 | 1400 | 34 | | | + |
| 5 | 550 | 72 | | | + |
| 15 | 198 | 90 | | | + |
| 45 | 20 | 99·9 | | | + |
| Control | | | 1960 | — | 0 |
| 200 | | | 2250 | 0 | 0 |
| 400 | | | 1630 | 23 | −6 |
| 800 | | | 873 | 58·6 | −18 |

Each group contained eight animals

$$\frac{ED_{50}\text{ D-Yoshida s.c. sarcoma} = 3500\text{ mg. per kg.}}{ED_{50}\text{ Yoshida s.c. sarcoma} = 13\cdot5\text{ mg. per kg.}} = 260$$

Thus the degree of resistance is 260.

*Cross-resistance tests on Yoshide s.c. sarcoma and on DBM-resistant D-Yoshida s.c. sarcoma*

The degree of inhibition obtained with the two types of tumours were compared in both cases with their own controls. The level of significance was calculated by comparing the numerical value of inhibition obtained with D-Yoshida sarcoma to the inhibition obtained on the sensitive tumour.

The data of Table II provides evidence of the resistance of D-Yoshida sarcoma to a variety of biological alkylating agents and also to Mitomycin C. The tumour however, retained its sensitivity to antimetabolites and mitotic poisons.

### DISCUSSION

1,6-Dibromo-1,6-dideoxy-D-mannitol/DBM/represents a new type of compound in the family of biological alkylating agents, as its molecule contains none of the known biological alkylating moieties, such as dichloroethylamine, ethyleneimine, mesyl or epoxide groups.

We described in our earlier papers (Institoris *et al.*, 1961; Institoris and Horváth 1964) that as far as its action is concerned DBM seems to be similar to 1,6-dimesyl-mannitol. Its haematological effect is directed mainly to the myeloid system.

In our studies of the action-structure relationships we have shown (Institoris *et al.*, 1964) that for the action of DBM not only the pair of Br atoms are important but the whole of the dibromo-mannitol molecule plays a decisive role. Thus the compound cannot be considered as an analogue of dimesyl-mannitol. In the competitive tests, the differences between DBM and other alkylating agents affecting the myeloid line were revealed (Csányi, 1964). It was therefore necessary to perform cross-resistance tests.

TABLE II.—*Cross-resistance Tests on Yoshida s.c. Sarcoma and onDBM Resistant D-Yoshida s.c. Sarcoma*

| Agent | Dose mg/kg 5 × i.p. | Yoshida s.c. sarcoma: Weight of tumour mg. | Yoshida s.c. sarcoma: Inhibition % | P* | D-Yoshida s.c. sarcoma: Weight of tumour mg. | D-Yoshida s.c. sarcoma: Inhibition % | Change in body-weight % |
|---|---|---|---|---|---|---|---|
| Control | — | 2490 ± 280 | — | — | 3440 ± 592 | — | +5 |
| DBM | 70 | 25 ± 11 | 99·1 | 0·01 | 2600 ± 420 | 24 | +1 |
| Mannitol Myleran | 70 | 22 ± 10 | 99·1 | 0·01 | 1570 ± 154 | 54·3 | +2 |
| Myleran | 10 | 10 ± 5 | 99·6 | 0·01 | 1350 ± 265 | 60·8 | −3·5 |
| Dibromdulcitol | 20 | 10 ± 2 | 99·6 | 0·01 | 2130 ± 457 | 41 | +2 |
| Degranol | 10 | 1010 ± 9 | 99·6 | 0·01 | 1720 ± 281 | 50 | −5 |
| Mitomycin-C | 0·5 | 12 ± 2 | 99·5 | 0·01 | 1340 ± 309 | 61 | −13 |
| Fluorouracyl | 20 | 930 ± 423 | 63·0 | 0·5 | 1550 ± 472 | 55 | −2† |
| Control | — | 2180 ± 146 | — | — | 1341 ± 121 | — | +1 |
| Mitomen | 1 | 158 ± 14 | 90·0 | 0·01 | 1204 ± 124 | 34 | +3·7 |
| Methotrexate | 0·2 | 76 ± 8 | 97·0 | 0·51 | 56 ± 8 | 95·8 | +15 |
| Vinblastine | 0·2 | 262 ± 32 | 89·0 | 0·5 | 158 ± 15 | 88·6 | +4 |
| Control | — | 8270 ± 790 | — | — | 2467 ± 320 | — | +40 |
| Bayer E-39 | 0·2 | 20 ± 3 | 99·9 | 0·01 | 2150 ± 202 | 12 | +21 |
| Thio-TEPA | 2·0 | 30 ± 5 | 99·9 | 0·01 | 1480 ± 149 | 41 | +20 |
| Control | — | 3490 ± 360 | — | — | 2300 ± 142 | — | +16 |
| Leukeran | 1·0 | 630 ± 3 | 84·0 | 0·01 | 1794 ± 96 | 22 | +8 |
| R-74** | 1·0 | 226 ± 32 | 94·3 | 0·01 | 1790 ± 78 | 22 | +10 |
| Actinomycin-C | 0·07 | 2190 ± 142 | 44·5 | 0·5 | 827 ± 58 | 64 | +4 |
| Tris-mustard | 0·05 | 90 ± 18 | 98·0 | 0·01 | 2470 ± 328 | 0 | +11 |

* = compared to the inhibition on D-Yoshida sarc.
** = 1,4-di/mesyloxyethylamino″-1,4-dideoxy-m-erythritol dichlorhydrate
† = 2/8 death.

The results of these experiments provide clear cut evidence of the *alkylating* nature of DBM, though its mechanism of action has not been established with certainty. The DBM-resistant tumour was found to be resistant both to agents classified into the lymphoid-active class (Elson, 1958) mustard derivatives, ethyleneimines and to myeloid-active compounds (Myleran, Mannitol-Myleran), and was found resistant also to another halogen sugar alcohol, namely to dibromodulcitol. Similarly to other resistant substrains, this type of tumour was also resistant to Mytomycin C.

The failure of this tumour to become resistant to antimetabolite type compounds excludes the possibility of some antimetabolic-like action of the drug which anyway seemed unlikely by our earlier experiments. Our described results give further evidence of the common feature of alkylating agents, as far as its principal behaviour is concerned, in spite of its quite different chemical structure. Thus we can support the conclusions that the differences among individual effective biological alkylating agents may be in relation with their chemical and physiochemical properties and not directly related to their alkylating potency.

### SUMMARY

A new DBM-resistant Yoshida s.c. sarcoma strain has been developed by means of the continuous adaptation method.

The degree of resistance of the D-Yoshida s.c. sarcoma was established at the 20th generation. It has been found to be 260-fold.

Several cross-resistance tests have been carried out with various types of antineoplastic agents. The data of these investigations demonstrated the presence of resistance to a variety of biological alkylating agents and also to Mitomycin. The D-Yoshida sarcoma strain was, however, sensitive to antimetabolites and mitotic poisons. These data provide valuable evidence of the alkylating nature of 1,6-dibromo-1,6-dideoxy-d-mannitol(DBM), Myelobromol$^{R}$ Chinoin.

### REFERENCES

CSÁNYI, E.—(1964) Paper at the Xth *Int. Congr. Haemat.*, Stockholm.—(1965) *Arzneimittel-Forsch.*, **15**, 198.

CSÁNYI, E., HORVÁTH, P. AND INSTITORIS, L.—(1964) *Arzneimittel-Forsch.*, **14**, 670.

CSÁNYI, E., HORVÁTH, P., INSTITORIS, L. AND VARGHA, L.—(1961) V. Ung. Krebstagungen, Budapest Akadémia, 1962, Budapest.

ECKHARDT, S., INSTITORIS, L., HORVÁTH, P., MEDGYES, A., MASSZI, F., HARTAI, F. AND HINDY, I.—(1964) *Orv. Hétil.*, **105**, 547.

ECKHARDT, S., SELLEI, C., HORVÁTH, P. AND INSTITORIS, L.—(1963) *Cancer Chemother. Rep.*, **33**, 57.

ELSON, L. A.—(1958) *Ann N.Y. Acad. Sci.*, **68**, 826.

GÁTI, E.—(1965) Lecture at the VIIth Hung. Oncol. Congr., Budapest.

HIRONO, T.—(1954) *Nagoya J. med. Sci.*, **17**, 102.

HORVÁTH, P., INSTITORIS, L. AND CSÁNYI, E.—(1966) *Arzneimittel-Forsch.*, in press.

INSTITORIS, L. AND HORVÁTH, P.—(1964) *Arzneimittel-Forsch.*, **14**, 668.

INSTITORIS, L., HORVÁTH, P. AND CSÁNYI, E.—(1961) 'IInd Symp. int. Chemother. Neapel. Proceedings II', **250**, 1963. S. Karger, Basel, 1963.—(1964) *Neoplasma*, **11**, 245.

MYURA, Y.—(1961) *J. Biochem.*, **49**, 502.

MORIWAKI, A.—(1963) *Gann*, **54**, 323.

SAKURAI, Y.—(1963) *Nippon Rinsho Jap. J. clin. Med.*, **21**, 2372.

SELLEI, C.—(1961) 'IInd Symp. int. Chemother. Neapel. Proceedings III'. S. Karger, Basel, 1963.

SELLEI, C. AND ECKHARDT, S.—(1963) *Revue fr. Étud. clin. biol.*, **8**, 483.

UJHÁZY, V. AND WINKLER, A.—(1965) *Neoplasma*, **12**, 11.


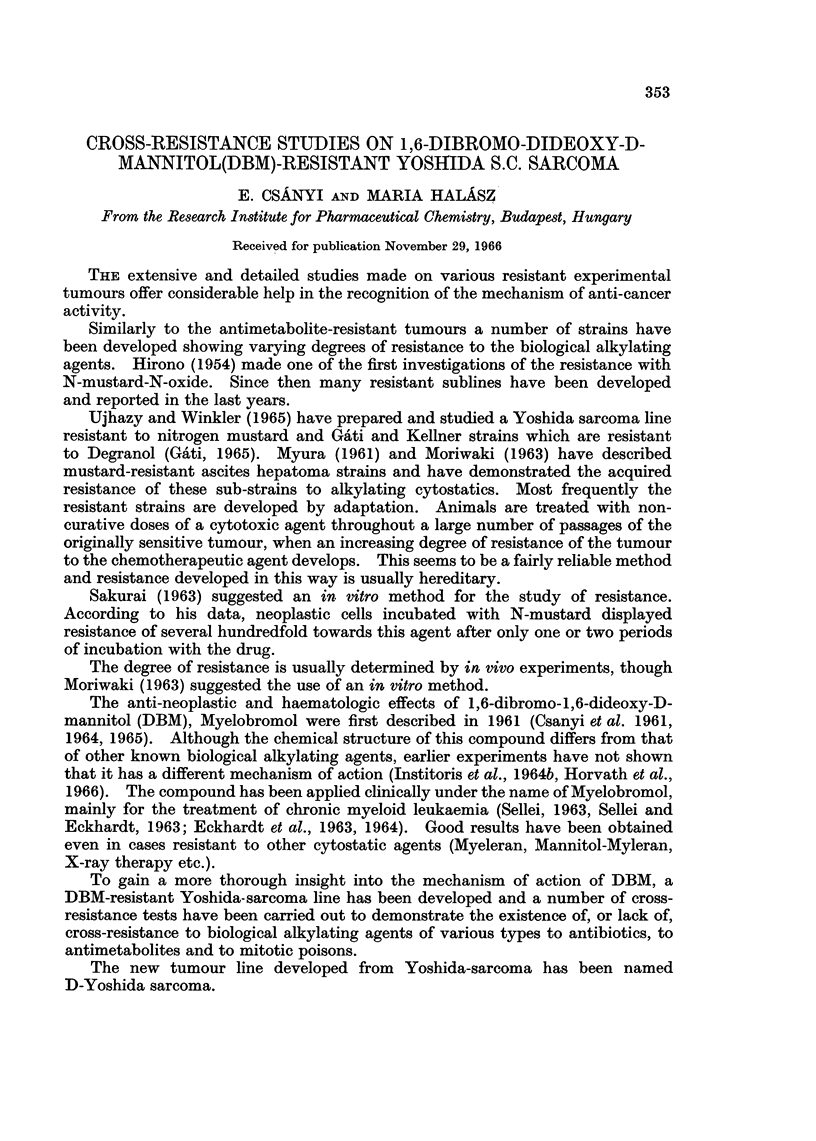

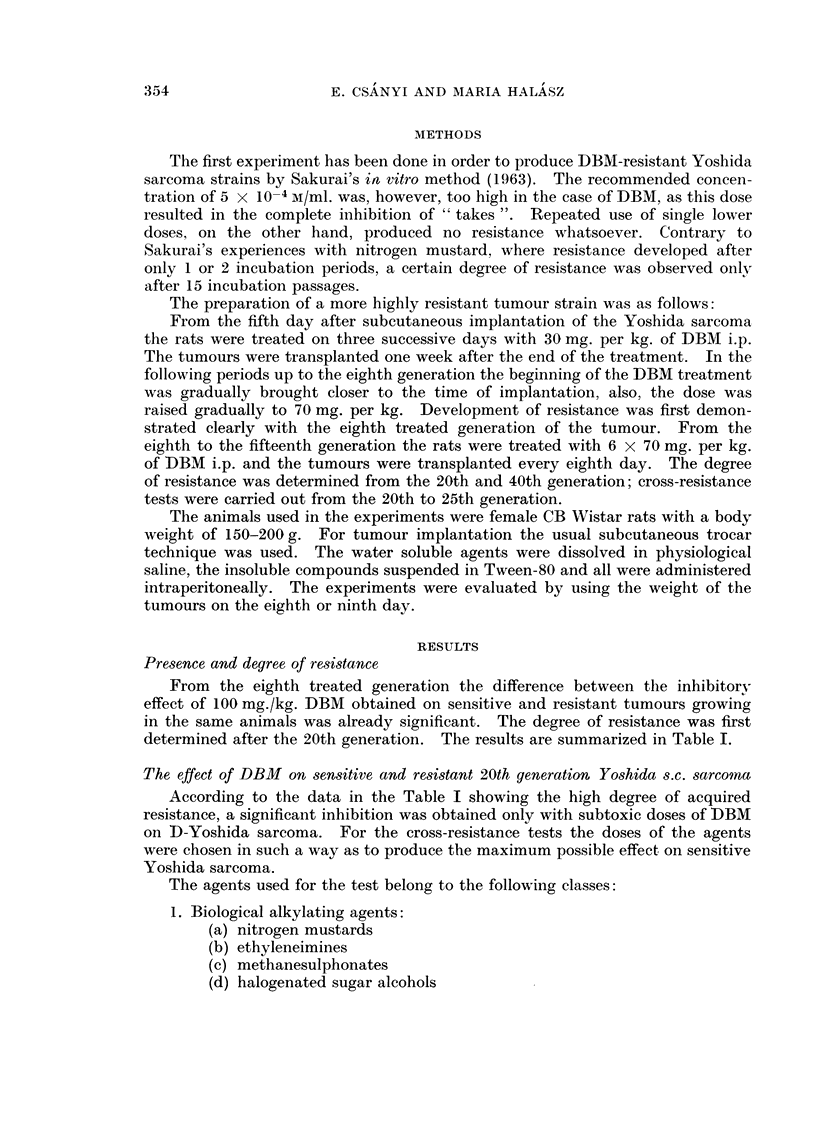

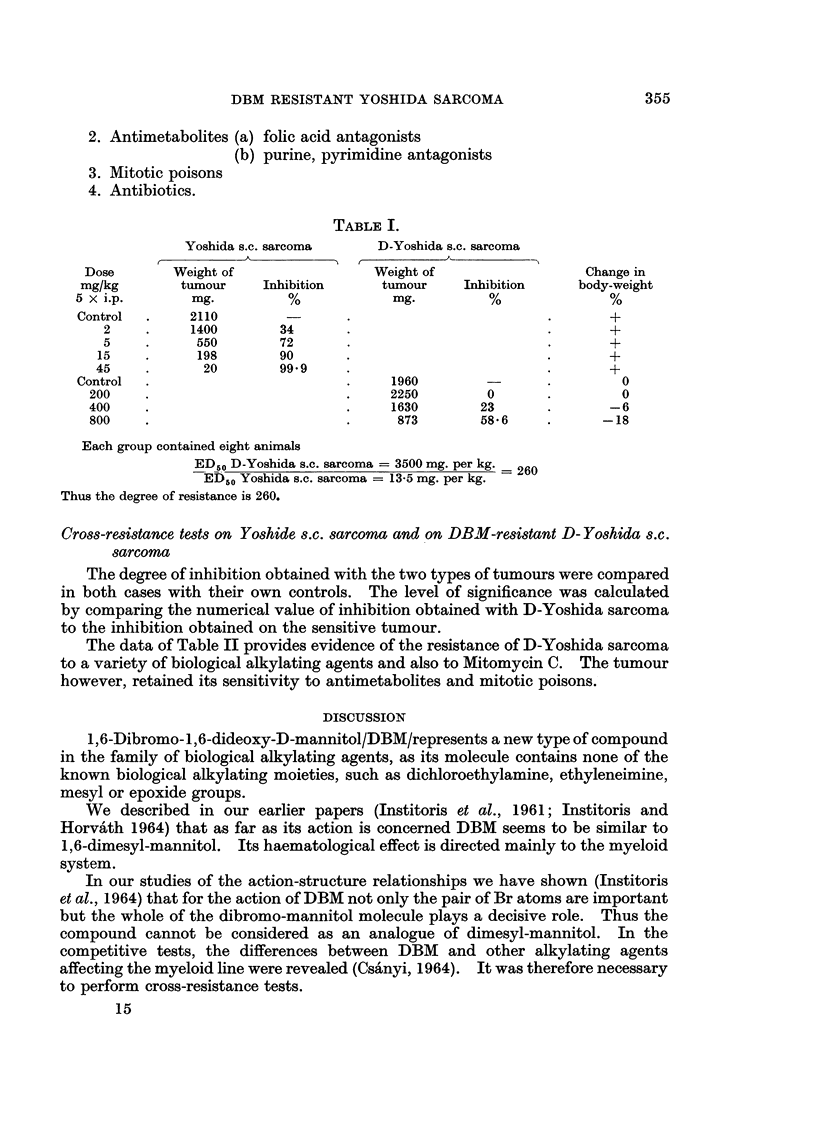

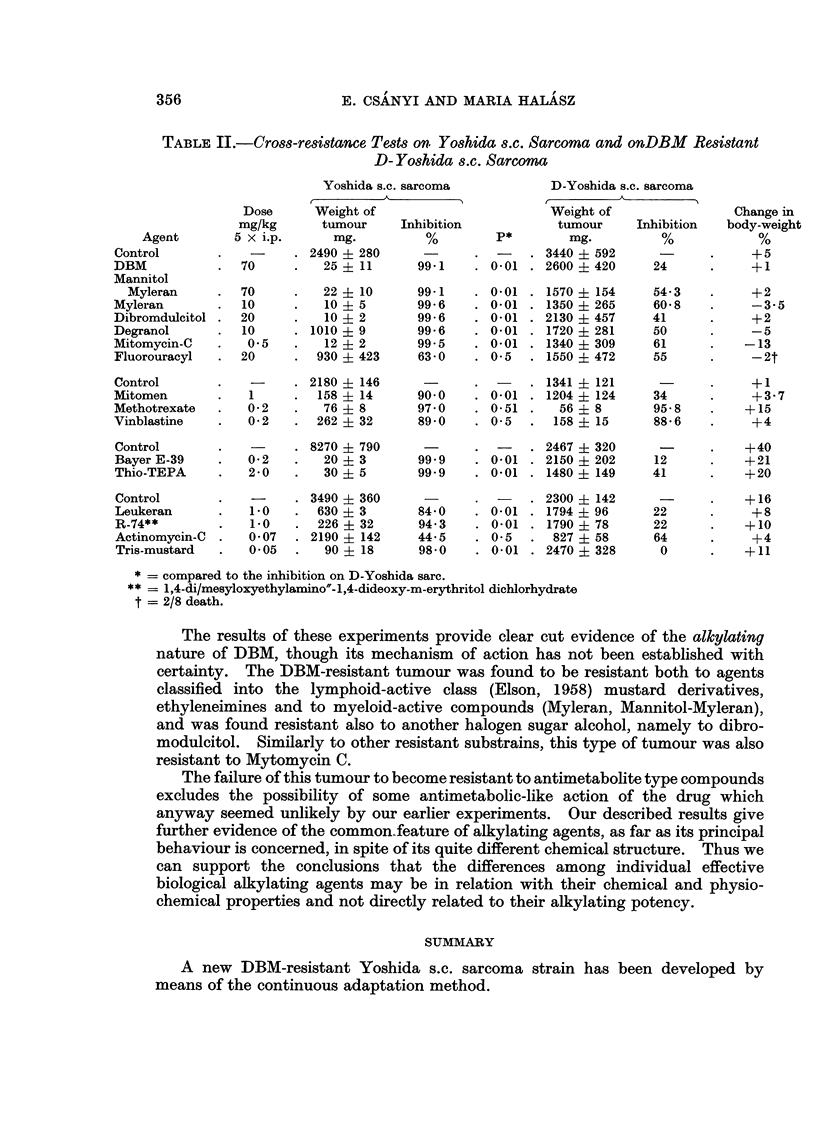

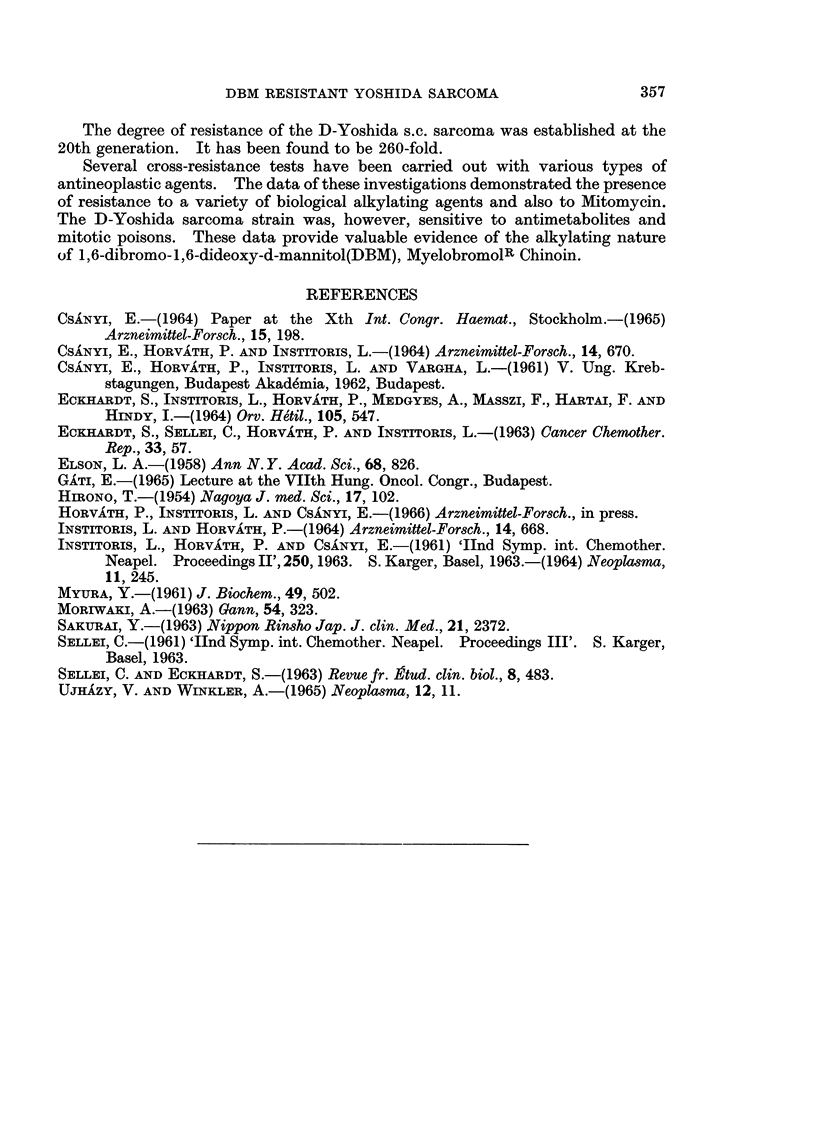


## References

[OCR_00276] CSANYI E. (1965). DIE WIRKUNG EINES NEUEN CYTOSTATICUMS, DES 1,6-DIBROM-1,6-DIDESOXY-D-MANNITS, AUF DIE HAEMOPOESE DER RATTE.. Arzneimittelforschung.

[OCR_00286] ECKHARDT S., SELLEI C., HORVATH P., INSTITORISZ L., MEDGYES A., MASSZI F., HARTAI F., HINDY I. (1964). AZ 1,6-DIBROM-1,6-D-DIDESOXYMANNIT (DBM) HAT'ASA CHRONIKUS MYELOID LEUK'EMI'ABAN.. Orv Hetil.

[OCR_00294] ELSON L. A. (1958). Hematological effects of the alkylating agents.. Ann N Y Acad Sci.

[OCR_00280] INSTITORIS L., HORVATH P. I. (1964). UNTERSUCHUNGEN UEBER ZUSAMMENHAENGE ZWISCHEN ALPHA-OMEGA-SUBSTITUTION UND CYTOSTATISCHER AKTIVITAET BEI EINIGEN ZUCKERALKOHOL-DERIVATEN UND BEI 1,6-DIBROM-1,6-DIDESOXY-D-MANNIT.. Arzneimittelforschung.

[OCR_00300] INSTITORIS L., HORVATH P. I. (1964). UNTERSUCHUNGEN UEBER ZUSAMMENHAENGE ZWISCHEN ALPHA-OMEGA-SUBSTITUTION UND CYTOSTATISCHER AKTIVITAET BEI EINIGEN ZUCKERALKOHOL-DERIVATEN UND BEI 1,6-DIBROM-1,6-DIDESOXY-D-MANNIT.. Arzneimittelforschung.

[OCR_00316] SELLEI C., ECKHARDT S. (1963). [Trial treatment of neoplastic diseases by 1,6-dibromomannitol].. Rev Fr Etud Clin Biol.

